# A First Look at ARFome: Dual-Coding Genes in Mammalian Genomes

**DOI:** 10.1371/journal.pcbi.0030091

**Published:** 2007-05-18

**Authors:** Wen-Yu Chung, Samir Wadhawan, Radek Szklarczyk, Sergei Kosakovsky Pond, Anton Nekrutenko

**Affiliations:** 1 Center for Comparative Genomics and Bioinformatics, The Pennsylvania State University, University Park, Pennsylvania, United States of America; 2 Integrative Bioinformatics Institute, Vrije Universiteit, Amsterdam, The Netherlands; 3 Antiviral Research Center, University of California San Diego, La Jolla, California, United States of America; University of Chicago, United States of America

## Abstract

Coding of multiple proteins by overlapping reading frames is not a feature one would associate with eukaryotic genes. Indeed, codependency between codons of overlapping protein-coding regions imposes a unique set of evolutionary constraints, making it a costly arrangement. Yet in cases of tightly coexpressed interacting proteins, dual coding may be advantageous. Here we show that although dual coding is nearly impossible by chance, a number of human transcripts contain overlapping coding regions. Using newly developed statistical techniques, we identified 40 candidate genes with evolutionarily conserved overlapping coding regions. Because our approach is conservative, we expect mammals to possess more dual-coding genes. Our results emphasize that the skepticism surrounding eukaryotic dual coding is unwarranted: rather than being artifacts, overlapping reading frames are often hallmarks of fascinating biology.

## Author Summary

A textbook human gene encodes a protein using a single reading frame. Alternative splicing brings some variation to that picture, but the notion of a single reading frame remains. Although this is true for most of our genes, there are exceptions. Like viral counterparts, some eukaryotic genes produce structurally unrelated proteins from overlapping reading frames. The examples are spectacular (G-protein alpha subunit [*Gnas1*] or *INK4a* tumor suppressor), but scarce. The scarcity is anthropogenic in origin: we simply do not believe that dual-coding genes can occur in eukaryotes. To challenge this assumption, we performed the first genome-wide scan for mammalian genes containing alternative reading frames located out of frame relative to the annotated protein-coding region. Using a newly developed statistical framework, we identified 40 such genes. Because our approach is very conservative, this number is likely a significant underestimate, and future studies will identify more alternative reading frame–containing genes with fascinating biology.

## Introduction

Any stretch of DNA contains six reading frames and can potentially code for multiple proteins. Situations when two partially overlapping reading frames code for functional polypeptides (dual coding) are quite common in bacteriophages and viruses (e.g., φX174, HIV-1, hepatitis C, or influenza A), where constraints on the genome size are strict. On the other hand, dual coding in vast eukaryotic genomes was reported to be scarce and restricted to short regions with secondary reading frames having poor phylogenetic conservation [1].

**Figure 1:**
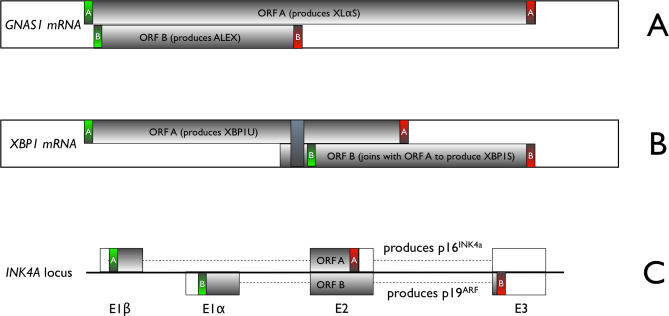
Three Known Examples of Mammalian Dual-Coding Genes (A) A transcript of the *Gnas1* gene contains two reading frames and produces two structurally unrelated proteins, XLαs and ALEX, by differential utilization of translation start sites. (B) A newly transcribed *XBP1* mRNA can only produce protein XBP1U from ORF A. Removal of a 26-bp spacer (yellow rectangle) joins the beginning of ORF A with ORF B and translates into a different product called XBP1S. (C) *Ink4a* generates two splice variants that use different reading frames within exon E2 to produce the proteins p16^Ink4a^ and p19^ARF^ (exon names as in [8]).

Yet, three known human genes (*GNAS1, XBP1,* and *INK4a*; Figure 1) defy this pattern by having long, well-conserved dual-coding regions (e.g., dual-coding region in XBP1 is conserved from worms to mammals [2]). In addition, the three cases exemplify some of the most striking biological phenomena and invite us to look at dual coding in greater detail. In *GNAS1,* a single transcript simultaneously produces the alpha subunit of G-protein from the main reading frame, and a completely different protein, ALEX, using a +1 frame [3]. A transcript of *XBP1* can produce only a single protein at a time and uses the endonuclease IRE1 to switch between two overlapping reading frames [4]. *INK4a* generates two alternative transcripts that use different reading frames of a constitutive exon for translation to tumor suppressor proteins p16^INK4a^ and p14^ARF^ [5]. Although *GNAS1, XBP1,* and *INK4a* are drastically different, there are striking parallels in the way they function. Products of the main and alternative reading frames perform related tasks, either by binding and regulating each other (*GNAS1* and *XBP1*), or by complementing each other in performing a common function *(INK4a)* [6–8].

Dual coding is a costly arrangement because it limits the flexibility of amino acid composition [9]. A silent change in one frame is almost always guaranteed to be amino acid changing in the other. Although counterintuitive, this codependency may in fact lead to an increase of the apparent substitution rate when two frames become locked in an evolutionary race of compensatory changes. A chief example of this is the mammalian *GNAS1* locus, where the overlapping reading frames accumulate substitutions so fast that primate and rodent sequences become virtually unalignable [10]. Yet despite this cost, the dual coding in *GNAS1, XBP1,* and *INK4a* is preserved throughout mammalian taxa [10,11]. Are overlapping reading frames a new avenue for encoding functionally linked proteins?

## Results/Discussion

### Dual Coding Is Virtually Impossible by Chance

Before describing our analyses, we define terms used in this paper. A dual-coding gene contains two frames read in the same direction: canonical (annotated as protein coding in literature and/or databases) and alternative. The alternative reading frame (ARF) is shifted forward one or two nucleotides relative to the canonical frame (+1 and +2 ARFs, respectively). To identify dual-coding genes, we used a comparative genomics strategy, because all presently known alternative reading frames are conserved in multiple species. For example, ARFs in *Gnas1, XBP1,* and *INK4A* are conserved in all sequenced mammals [8,10,12].

To reliably find new dual-coding genes, we must determine how likely they are to occur by chance. Simulations designed to answer this question show that dual coding is statistically unlikely, suggesting that if overlapping coding regions are detected in orthologous sequences, they have a high chance of being truly functional. To determine a length threshold for identification of dual-coding regions (what is the longest dual-coding region that can arise by chance?), we conducted the following experiment. First, we generated alignments between 14,159 orthologous canonical reading frames from human and mouse transcripts (sequences, canonical frame boundaries, and orthology assignments were obtained from the Ensembl database at http://www.ensembl.org). We chose these two species because they have the highest number of annotated transcripts. Next, we “disassembled” all 14,159 human/mouse alignments into codon columns. By randomly picking codon columns from the previous step, we generated 10,000 simulated alignments with 5,000 columns each. Finally, we scanned simulated alignments for the presence of ARFs and built a length distribution (Figure S1). Only 0.1% of +1 ARFs were ≥500 bp, while none of the +2 ARFs extended beyond this threshold (the longest was 492 bp in the simulation).

A possible weakness of this approach is the assumption of codon independence, for it is well-known that protein-coding regions possess Markovian properties [13]. To address this issue, we conducted codon-based phylogenic parametric simulations, which do not break open reading frames (ORFs), and estimated codon frequencies from gene alignments with at least three taxa, which contained conserved, long +1 ARFs. Only 0.3% of simulated alignments preserved ARFs with 500 or more nucleotides (Figure S2). Thus, both simulations suggest that only a negligible amount of random dual-coding regions will reach 500 bp, and we set this length as the threshold for defining ARFs in orthologous coding regions.

### Defining Mammalian ARFs

**Figure 2:**
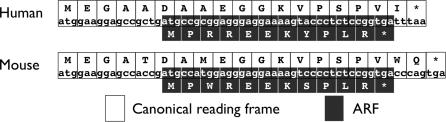
mRNAs from Human and Mouse Are Aligned Mouse mRNAs are indicated by lowercase letters. Each of the two mRNAs contains an annotated coding region (white boxes). Our algorithm looks for ARFs (black boxes) that are shifted one (shown) or two nucleotides relative to the annotated frame. The locations of the ARFs must be conserved between the species. Specifically, the ARFs in the two species must overlap for at least 500 bp.

Using 500 bp as the lower bound, we identified 149 ARFs that were conserved in human and mouse. An example is shown in Figure 2 (see Figures S3 and S4 for procedure steps and detection of ARFs from multiple alignments). Although all 149 candidate ARFs were conserved in the two species and were longer than the empirically derived threshold, some could still be false positives. For example, the amino acid sequence of the canonical protein may dictate specific codon composition, which in turn may render the nucleotide sequence of the canonical frame such that an ARF can be relatively long simply as an artifact of the codon usage pattern (e.g., having low complexity regions, or avoiding “problem” codons; see Table S2). To remove potential false positives, we developed the codon column replacement test (CCRT; see Materials and Methods). CCRT estimates how likely a given alignment is to contain an ARF by chance. If an ARF has a CCRT score of ≤5%, it is considered a reliable prediction. From the total of 149 ARFs, 66 satisfied this criterion. To make our final set even more conservative, we considered only those of the 66 ARFs that were conserved in at least one other species (rat and/or dog) in addition to human and mouse. The conservation requirement reduced the final set to 40 ARF-containing transcripts, which we examined in detail (Table 1). Note that our criteria are very conservative because (1) a number of true ARFs may be shorter than 500 bp (261 bp and 210 bp in *XBP1* and *Ink4A,* respectively) and (2) transcript data for dog and rat are incomplete, which may have led to the exclusion of some true ARFs. Genomic location of the ARFs are provided in Table S4 and can be visualized as a custom track at the University of California Santa Cruz Genome Browser [14] (a link is provided at http://nekrut.bx.psu.edu). Table S3 lists assignment of ARF-containing genes to Gene Ontology categories.

**Table 1:**
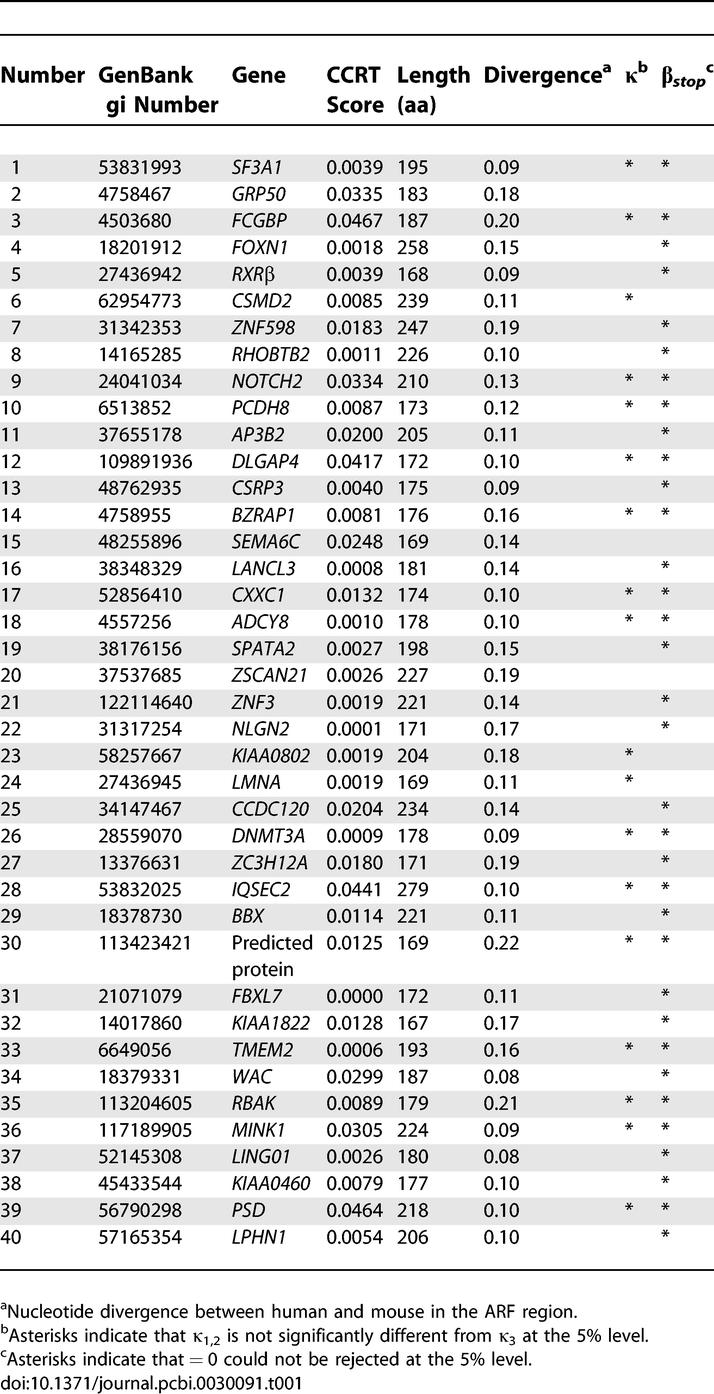
ARF-Containing Genes Identified Using a High-Stringency Approach

### Analysis of Nucleotide Substitutions Suggests Functionality of ARFs

Previous studies of ARF-containing genes showed that the region of overlap between canonical and alternative reading frames evolves under unique sets of constraints. If both proteins (encoded by canonical and alternative frames) are functional and maintained by purifying selection, the codependency between codon positions would manifest itself in a nucleotide substitution pattern that is sharply different from the one expected in single coding regions [10,11]. The difference in patterns can be used to test whether the dual-coding genes identified in our study are real. We developed two new approaches for the analysis of nucleotide substitutions—a codon substitution model for overlapping reading frames and a transition/transversion ratio test—to narrow the list of potential dual-coding genes to 15 high-confidence candidates. The codon model estimates five substitution rates for the overlapping reading frames by considering all 64 possible codon contexts for each one-nucleotide codon substitution in a given frame, and weighting each context based on its relative frequency in the extant sequences (see Materials and Methods). One of the rates, *β_STOP_*, which measures the propensity of substitutions in one frame toward introduction of stop codons in the other frame, is especially useful for testing the reliability of ARF predictions. This quantity measures the admissibility of stop codon–inducing contexts in the evolutionary past of the sample and is zero or near zero in functional ARFs. For example, when applied to biochemically characterized ARFs in *Gnas1* and *XBP1,* the hypothesis of *β_STOP_* being exactly zero cannot be rejected (*p* = 0.5 from likelihood ratio test). For 34 candidates, the hypothesis *β_STOP_* = 0 could not be rejected. From a series of parametric simulations we estimated that at *p* = 0.05, the test fails to reject the null hypothesis for 6% of the datasets that were simulated using a single reading frame model.

To confirm our results using an independent nucleotide-based approach (as opposed to the codon-based test described earlier), we applied the transition/transversion (*κ*) ratio test to make inferences about biological significance of ARFs. The test is based on the following reasoning: in most standard protein-coding regions (with only one reading frame), *κ* at the third codon position (*κ*_3_) is significantly different (higher) than at the first and second codon positions (*κ*_12_), so that *κ*_12_ < *κ*_3_ [15]. This is because most substitutions at the third codon position are synonymous, whereas in the first codon position all but eight substitutions are nonsynonymous, and all substitutions in the second codon position are nonsynonymous. By contrast, in overlapping reading frames, codon positions are codependent. For example, in a +1 ARF, the third codon positions correspond to the first codon positions of the canonical frame. Thus, almost every change in the third codon position of the ARF is guaranteed to change amino acids encoded in the canonical frame. However, if the ARF encodes a truly functional product, purifying selection would resist such changes, and the condition *κ*_12_ < *κ*_3_ would not hold. This gives us the opportunity to test functionality of ARF in our dataset by contrasting two hypotheses: *H_0_*: *κ*_12_ = *κ*_3_ (ARF does encode functional polypeptide) and *H_A_*: *κ*_12_ < *κ*_3_ (ARF *does not* encode functional polypeptide). To perform this test, we used a maximum likelihood framework to test *κ*_12_ and *κ*_3_ for equality [16]. Application of the test to our list of dual-coding genes identified 18 candidates.

Intersecting the results of the tests yielded 15 dual-coding genes as high-confidence candidates. The small number of species used in this study (four; a currently unavoidable limitation given the low annotation quality of mammalian genomes) limits the statistical power of our analyses and explains why the other candidates did not pass this test. Similar analyses of *Gnas1* and *XBP1* genes used eight or more sequences [10,11]. Adding more sequences, which should be possible in the near future, will increase the number of high-confidence candidates.

### What May Be the Potential Function of ARF-Encoded Proteins?

Although experimental confirmation of protein expression and genetic studies will ultimately answer this question, analysis of current literature provided us with clues to potential ARF functions. For example, one of the candidates is adenylate cyclase (ADCY8; Table 1), a membrane-bound enzyme that catalyses the formation of cyclic AMP from ATP [17]. A 534 bp ARF is located in the 5′-end of the *ADCY8* transcript. The corresponding region of the canonical peptide has two distinct functions: it interacts with Ca^2+^/calmodulin and binds to the catalytic subunit of protein phosphatase 2A (PP2a; [18]). Such “multitasking” is one of the features of dual-coding genes, where separate functions are performed by products of canonical frames and ARFs [7,8,19]. Two nucleotide substitutions affecting the amino acid sequence of ADCY8, W38A, and S66D (produced by mutagenesis) have conspicuous effects on ARF structure and calmodulin binding. W38A creates a stop in the ARF and disrupts calmodulin binding, but has no effect on association with PP2a. On the other hand, S66D does not disrupt ARF and has no effect on either calmodulin or PP2a binding [20]. Because in at least two instances products of ARF bind to the product of the canonical frame (i.e., *Gnas1* [6] and *XBP1* [7]), we speculate that the polypeptide encoded by the ARF may mediate the binding of calmodulin by ADCY8. In fact, ADCY8 has a number of unidentified protein interaction partners from yeast two-hybrid screen experiments, one of which may be the ARF-encoded polypeptide [18].

Another gene in our set, Misshapen/Nck-related Kinase (*MINK1*; see Table 1), is involved in a number of functions related to cell spreading, fiber formation, and cell-matrix adhesion. *MINK1* regulates the Jun kinase pathway (JNK) [21], is involved in thymocyte selection, and interacts with a large number of proteins controlling cytoskeletal organization, cell cycle, and apoptosis [22]. The MINK1 protein contains three functional domains (N-terminal kinase, intermediate, and C-terminal germinal center kinase) and exists as five distinct isoforms translated from alternatively spliced transcripts. All five transcripts contain an intact ARF, which covers the entire length of the intermediate domain. Extreme multifunctionality of MINK1 suggests that the ARF-encoded protein may be responsible for some of the functions. In addition, the intermediate region of the protein is the most variable in cross-species comparisons [23]. This provides additional support to the functionality of MINK1′s ARF: regions containing overlapping reading frames encoding functional proteins are likely to evolve faster in comparison with single-coding regions [10,11].

Retinoid X receptor beta (RXRβ; see Table 1) is a member of the retinoid X nuclear receptors that control transcription of multiple genes. In mice, RXRβ binds to the enhancer controlling major histocompatibility class I genes [24]. It is the only gene in our set in which the existence of the ARF was reported in the literature as an alternative N-terminus generated via alternative splicing [25], although this gene failed to pass our transition-to-transversion ratio test. Analysis of transcripts available for this gene shows that this was caused by the skipping of the second coding exon. Because the length of the skipped exon is not in multiples of three, this event switches the reading frame downstream of the splicing point. To recover the phase of the reading frame past the splicing point, the translation must be initiated at the ARF start codon. Because both transcripts (with and without a second exon) have identical 5′ ends, it is likely that the ARF is translated from the full-length transcript.

### Conclusions

Maintenance of dual-coding regions is evolutionarily costly and their occurrence by chance is statistically improbable. Therefore, an ARF that is conserved in multiple species is highly likely to be functional. Historically, dual-coding regions were largely overlooked as they violated the accepted views of the eukaryotic gene organization. For example, although the fact that *XBP1* produces two proteins was known for years, only one of them was considered biologically important. The confirmation for the function of the second protein came only recently, when three groups described its roles [7,19,26]. Dual coding is also difficult to confirm experimentally and computationally. For example, one cannot use expressed sequence tags (ESTs) to confirm expression of ARFs because in the cases described here, the same transcript expresses both proteins via the use of alternative translation starts. Using initiation codon context or protein structure predictions are not guaranteed to confirm or refute ARF functionality either: the most impressive example of dual coding, *Gnas1,* has poorly defined Kozak motifs [27] and produces proline-rich polypeptides without clearly defined secondary structure elements [3]. However, analyses of confirmed dual-coding regions allowed us to highlight unique properties and to use them in a genome-wide scan that identified 40 candidates.

Is this too much or too little? We emphasize that our criteria were set to be very strict to eliminate the noise. Therefore, the seemingly small number of candidates is likely just a subset of a larger “ARFome.” First, some ARFs are shorter than the stringent length threshold of 500 bp that we have set to eliminate most false positives. For example, the length of the dual-coding region in human *XBP1* is 261 bp [28], and is 210 bp in human *INK4a* [5]. Second, because only four species were included in the analyses of nucleotide substitutions, some dual-coding regions failed codon-based and transition/transversion ratio tests due to the lack of statistical power. As the annotation quality of other mammalian genomes increases, it will be possible to add more sequences into our analyses. Third, we required ARFs to be conserved in multiple species. A recent study has demonstrated that many dual-coding regions are specific to a narrow phylogenetic group (i.e., primates [1]) and would not be detected by the current implementation of our method. None of the 40 genes identified in our study overlaps with Liang and Landweber's dataset [1], as these authors primarily focused on short dual-coding regions arising from alternative splicing events. Finally, our approach assumes that the two proteins encoded by the dual-coding region evolve under a purifying selection regime as in all presently known mammalian dual-coding genes. This assumption was shown not to hold for some dual-coding regions of bacterial genomes [29]. Thus, 40 candidates is likely an underestimate. Improving annotation of additional mammalian species will allow us to conduct lower-stringency scans to define the size of the ARFome.

Our study provides a robust statistical framework for detection and computational validation of dual-coding regions. This methodology will work equally well in genome-wide screens (this study) and in situations in which an ARF in a single gene needs to be evaluated. Take another look at your gene; you might find an unexpectedly simple explanation, a second protein from the alternative reading frame, for experimental results that are otherwise difficult to interpret.

## Materials and Methods

### CCRT algorithm.

CCRT estimates how likely an alignment is to contain an ARF by chance. The algorithm works as follows. Consider an alignment of human and mouse protein-coding regions similar to that shown in Figure 2. It contains two reading frames: canonical (ORF, white) and alternative (ARF, black). The objective of CCRT is to test whether the ARF is or is not the artifact of nucleotide composition imposed by the ORF. CCRT takes two inputs: the alignment we just discussed and a codon column frequency table. The codon column frequency table is similar to a codon usage table but instead of codons, it contains alignments of codons from at least two species (in our case, human and mouse). The codon column frequency table is generated by first aligning all possible orthologous protein-coding regions between two (or more) species, splitting these alignments into individual codon alignments, and counting the frequency of each codon alignment. For this study, the table was constructed by aligning ~9,000 orthologous protein-coding regions from human and mouse (alignments can be downloaded from http://nekrut.bx.psu.edu).

Given an alignment and the codon frequency table, CCRT generates multiple simulated alignments (in this study we used 10,000 replicates) by replacing the original codon columns of the alignment with ones drawn from the codon column frequency table so that the amino acid translation is preserved in the ORF. The probability of drawing a codon alignment from the codon column frequency table is proportional to its frequency. The ORF translations of all simulated translations are identical to the ORF translation of the original alignment, but are guaranteed to be different at the nucleotide level. Finally, each simulated alignment is translated in the ARF, and the number of alignments with the full-length ARF is recorded. This number serves as the empirical *p*-value. A low *p-*value (<5%) indicates that a small fraction of simulated alignments contain ARFs, and therefore the ARF is not an artifact of nucleotide composition imposed by ORFs and can be considered a true ARF.

### Codon model for overlapping reading frames.

Consider an alignment of *N* codon sequences on *S* codons, which encodes two overlapping reading frames. We present the case in which the frames are shifted by one nucleotide relative to one another, but other cases can be handled by straightforward modifications. We refer to the two reading frames as *F0* (frame 0) and frame *F1* (frame +1). We also make use of the following notation: *π^ab^_ij_* denotes the frequency of dinucleotide **ij** in *a* and *b* codon positions (relative to *F0*) and *π^c^_k_* denotes the frequency of nucleotide **k** in the *c-*th codon position. These quantities are estimated by observed counts from a given alignment.

First, we define the model for codon evolution in *F0*. We discriminate four types of codon substitutions: *SS* (synonymous in both frames), *SN* (synonymous in *F0* and nonsynonymous in *F1*), *NS* (nonsynonymous in *F0* and synonymous in *F1*), and *NN* (nonsynonymous in both frames). We model the process of character substitution using a Markov process operating on codons and defined by the instantaneous rate matrix **Q**. Following the common practice of allowing nonzero rates for single instantaneous nucleotide substitutions only, we assign substitution rates *α* to all one-nucleotide SS substitutions, β_01_ to *SN* substitutions, β_10_ to *NS* substitutions, and β_11_ to *NN* substitutions. In addition, we introduce another rate—β_*STOP*_—for all those substitutions that introduce a stop codon in one of the two frames. Because the evolution at a given position in *F0* depends on the flanking nucleotides (two upstream and one downstream), we condition the substitutions at a codon in *F0* on the values of the relevant nucleotides, compute transition probabilities for each of the 64 possibilities, and weight over the frequency distributions *π* ^12^ and *π* ^3^.

Formally, the instantaneous rate of substituting a nonstop codon **x** = *x*_1_ *x*_2_ *x*_3_ with a nonstop codon **y** = *y*_1_ *y*_2_ *y*_3_ in *F0* conditioned on the values of the two upstream nucleotides *u*_1_ *u*_2_ and the downstream nucleotide *d*_1_:

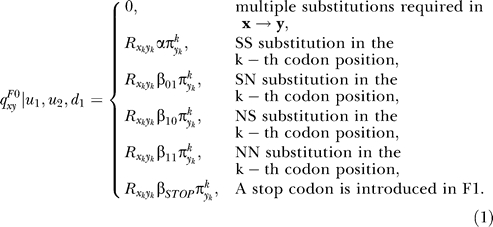
Conditioning on *u*_1_,*u*_2_,*d*_1_ is necessary to determine whether a substitution in *F0* results in a synonymous or a nonsynonymous change in *F1*. *R_nm_* denotes the rate of substitution for nucleotides *n* and *m* relative to that of *A* → *G*. We set *R_nm_* = *R_mn_* to ensure time reversibility. One can check that for any triplet *u*_1_,*u*_2_,*d*_1_, the equilibrium distribution of the Markov process defined by this rate matrix is

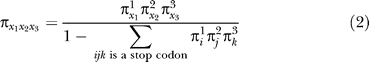
Second, we describe an analogous rate matrix 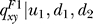 for *F1*. This rate matrix is conditioned on one upstream nucleotide *u*_1_ and two downstream nucleotides *d*_1_,*d*_2_.

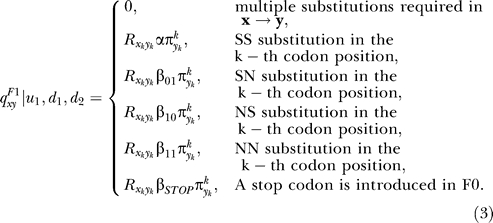
Transition matrices **T**(*t*) for the processes are matrix exponentials of **Q** *t*, for the appropriate rate matrix **Q**. For computational tractability, we assume that the evolution at codon *c* can be adequately described by computing the expectation over flanking upstream and downstream nucleotides. Specifically, if 
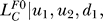
 is the phylogenetic likelihood at codon *c* in frame *F0*, conditioned on the flanking nucleotides, then the unconditional likelihood can be computed as

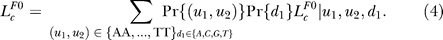
Analogous calculation can be performed for frame *F1*. Finally, we define the joint likelihood of the entire dataset (omitting the first and the last codons in *F0*) as

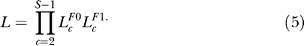



Parameter estimates such as branch lengths and substitution rates can be obtained by maximizing the likelihood as a function of model parameters with standard numerical optimization techniques. Due to the structure of the genetic code, most of the possible single-nucleotide substitutions lead to nonsynonymous changes in at least one of the reading frames (Table S1). To evaluate the evolutionary regime in a multiple reading frame alignment, we test the null hypothesis to evaluate whether the introduction of premature stop codons is disallowed. The test defined a one-sided constraint on a single parameter, and the significance can be evaluated using the likelihood ratio test with the approximate distribution of the test statistic.

## Supporting Information

Figure S1 Distribution of Lengths of Maximal ARFs Detected in 10,000 Simulated Alignments (70 KB PDF)

Figure S2 Distribution of Lengths of Maximal ARFs, Based on 35,000 Parametric Simulations Based on Codon Model Fits to Orthologous Gene Alignments from Three or Four species A total of 39 gene fits, each with at least 500 bp sampled equiprobably. Only 0.29% of simulated alignments had open ARFs with 500 or more nucleotides.
(29 KB PDF)

Figure S3 Number of Possible Dual-Coding Genes and Corresponding Criteria The number of possible dual-coding genes are shown in parentheses.
(40 KB PDF)

Figure S4 The Discovery and Definition of Conserved Dual-Coding Regions from Multispecies Alignments The orthologous transcripts from four species were first aligned and then translated using the second reading frame. Hence, additional start and stop codons appeared in the translation. For each of the species, an uninterrupted segment of peptides were identified (the dotted line with arrow ends in both directions), and the first start codon was marked. The region between the closest start–stop codons was defined as the ARF region. From the same set of transcripts, regions from the beginning to the first stop codon in any one of the species and the last stop codon to the end of the transcript were defined as flanking the ORF region.
(47 KB PDF)

Table S1 Proportion of Substitution Types (in Percent) in Each Codon Position of *F0* and *F1* Averaged over All Possible Nucleotide Contexts (38 KB PDF)

Table S2 Proportion (Percent) of Prefix and Suffix Codons (out of 3,721 Possibilities) That, for a Given Middle Codon, Do Not Induce a Stop Codon in the +1 Reading Frame Brighter colors indicate less-tolerated codons.
(42 KB PDF)

Table S3 Gene Ontology Categories of the 40 Candidate Genes (58 KB PDF)

Table S4 Genomic Coordinates of the 40 Candidate Genes (40 KB PDF)

## Abbreviations

ARF: alternative reading frame
CCRT: codon column replacement test
ORF: open reading frame

## References

[pcbi-0030091-b001] Liang H, Landweber LF (2006). A genome-wide study of dual coding regions in human alternatively spliced genes. Genome Res.

[pcbi-0030091-b002] Calfon M, Zeng H, Urano F, Till JH, Hubbard SR (2002). IRE1 couples endoplasmic reticulum load to secretory capacity by processing the XBP-1 mRNA. Nature.

[pcbi-0030091-b003] Klemke M, Kehlenbach RH, Huttner WB (2001). Two overlapping reading frames in a single exon encode interacting proteins—A novel way of gene usage. EMBO J.

[pcbi-0030091-b004] Yoshida H, Matsui T, Yamamoto A, Okada T, Mori K (2001). *XBP1* mRNA is induced by ATF6 and spliced by IRE1 in response to ER stress to produce a highly active transcription factor. Cell.

[pcbi-0030091-b005] Quelle DE, Zindy F, Ashmun RA, Sherr CJ (1995). Alternative reading frames of the *INK4a* tumor suppressor gene encode two unrelated proteins capable of inducing cell cycle arrest. Cell.

[pcbi-0030091-b006] Freson K, Jaeken J, Van Helvoirt M, de Zegher F, Wittevrongel C (2003). Functional polymorphisms in the paternally expressed XLalphas and its cofactor ALEX decrease their mutual interaction and enhance receptor-mediated cAMP formation. Hum Mol Genet.

[pcbi-0030091-b007] Yoshida H, Oku M, Suzuki M, Mori K (2006). pXBP1(U) encoded in *XBP1* pre-mRNA negatively regulates unfolded protein response activator pXBP1(S) in mammalian ER stress response. J Cell Biol.

[pcbi-0030091-b008] Sharpless NE (2005). *INK4a*/ARF: A multifunctional tumor suppressor locus. Mutat Res.

[pcbi-0030091-b009] Keese PK, Gibbs A (1992). Origins of genes: “Big bang” or continuous creation?. Proc Natl Acad Sci U S A.

[pcbi-0030091-b010] Nekrutenko A, Wadhawan S, Goetting-Minesky P, Makova KD (2005). Oscillating evolution of a mammalian locus with overlapping reading frames: An XLalphas/ALEX relay. PLoS Genet.

[pcbi-0030091-b011] Nekrutenko A, He J (2006). Functionality of unspliced *XBP1* is required to explain evolution of overlapping reading frames. Trends Genet.

[pcbi-0030091-b012] Schroder M, Kaufman RJ (2005). The mammalian unfolded protein response. Annu Rev Biochem.

[pcbi-0030091-b013] Burge C (1997). Identification of genes in human genomic DNA.

[pcbi-0030091-b014] Kent WJ, Sugnet CW, Furey TS, Roskin KM, Pringle TH (2002). The human genome browser at UCSC. Genome Res.

[pcbi-0030091-b015] Li WH (1997). Molecular evolution.

[pcbi-0030091-b016] Pond SL, Frost SD, Muse SV (2005). HyPhy: Hypothesis testing using phylogenies. Bioinformatics.

[pcbi-0030091-b017] Cooper DM (2003). Regulation and organization of adenylyl cyclases and cAMP. Biochem J.

[pcbi-0030091-b018] Crossthwaite AJ, Ciruela A, Rayner TF, Cooper DM (2006). A direct interaction between the N terminus of adenylyl cyclase AC8 and the catalytic subunit of protein phosphatase 2A. Mol Pharmacol.

[pcbi-0030091-b019] Shen X, Ellis RE, Sakaki K, Kaufman RJ (2005). Genetic interactions due to constitutive and inducible gene regulation mediated by the unfolded protein response in C. elegans. PLoS Genet.

[pcbi-0030091-b020] Smith KE, Gu C, Fagan KA, Hu B, Cooper DM (2002). Residence of adenylyl cyclase type 8 in caveolae is necessary but not sufficient for regulation by capacitative Ca(2+) entry. J Biol Chem.

[pcbi-0030091-b021] Hu Y, Leo C, Yu S, Huang BC, Wang H (2004). Identification and functional characterization of a novel human misshapen/Nck interacting kinase-related kinase, hMINK beta. J Biol Chem.

[pcbi-0030091-b022] Qu K, Lu Y, Lin N, Singh R, Xu X (2004). Computational and experimental studies on human misshapen/NIK-related kinase MINK-1. Curr Med Chem.

[pcbi-0030091-b023] Dan I, Watanabe NM, Kobayashi T, Yamashita-Suzuki K, Fukagaya Y (2000). Molecular cloning of MINK, a novel member of mammalian GCK family kinases, which is up-regulated during postnatal mouse cerebral development. FEBS Lett.

[pcbi-0030091-b024] Hamada K, Gleason SL, Levi BZ, Hirschfeld S, Appella E (1989). H-2RIIBP, a member of the nuclear hormone receptor superfamily that binds to both the regulatory element of major histocompatibility class I genes and the estrogen response element. Proc Natl Acad Sci U S A.

[pcbi-0030091-b025] Fleischhauer K, Park JH, DiSanto JP, Marks M, Ozato K (1992). Isolation of a full-length cDNA clone encoding a N-terminally variant form of the human retinoid X receptor beta. Nucleic Acids Res.

[pcbi-0030091-b026] Tirosh B, Iwakoshi NN, Glimcher LH, Ploegh HL (2006). Rapid turnover of unspliced xbp-1 as a factor that modulates the unfolded protein response. J Biol Chem.

[pcbi-0030091-b027] Kozak M (2001). Extensively overlapping reading frames in a second mammalian gene. EMBO Rep.

[pcbi-0030091-b028] Mori K (2003). Frame switch splicing and regulated intramembrane proteolysis: Key words to understand the unfolded protein response. Traffic.

[pcbi-0030091-b029] Rogozin IB, Spiridonov AN, Sorokin AV, Wolf YI, Jordan IK (2002). Purifying and directional selection in overlapping prokaryotic genes. Trends Genet.

