# Supplementary material for: A First Look at ARFome: Dual-Coding Genes in Mammalian Genomes
Source: PLoS Comput Biol. 2007 May 18;3(5):e91. doi: 10.1371/journal.pcbi.0030091 (PMC1868773; doi:10.1371/journal.pcbi.0030091)
Supplement: Supplementary file 1 [file pcbi.0030091.sg001.pdf]

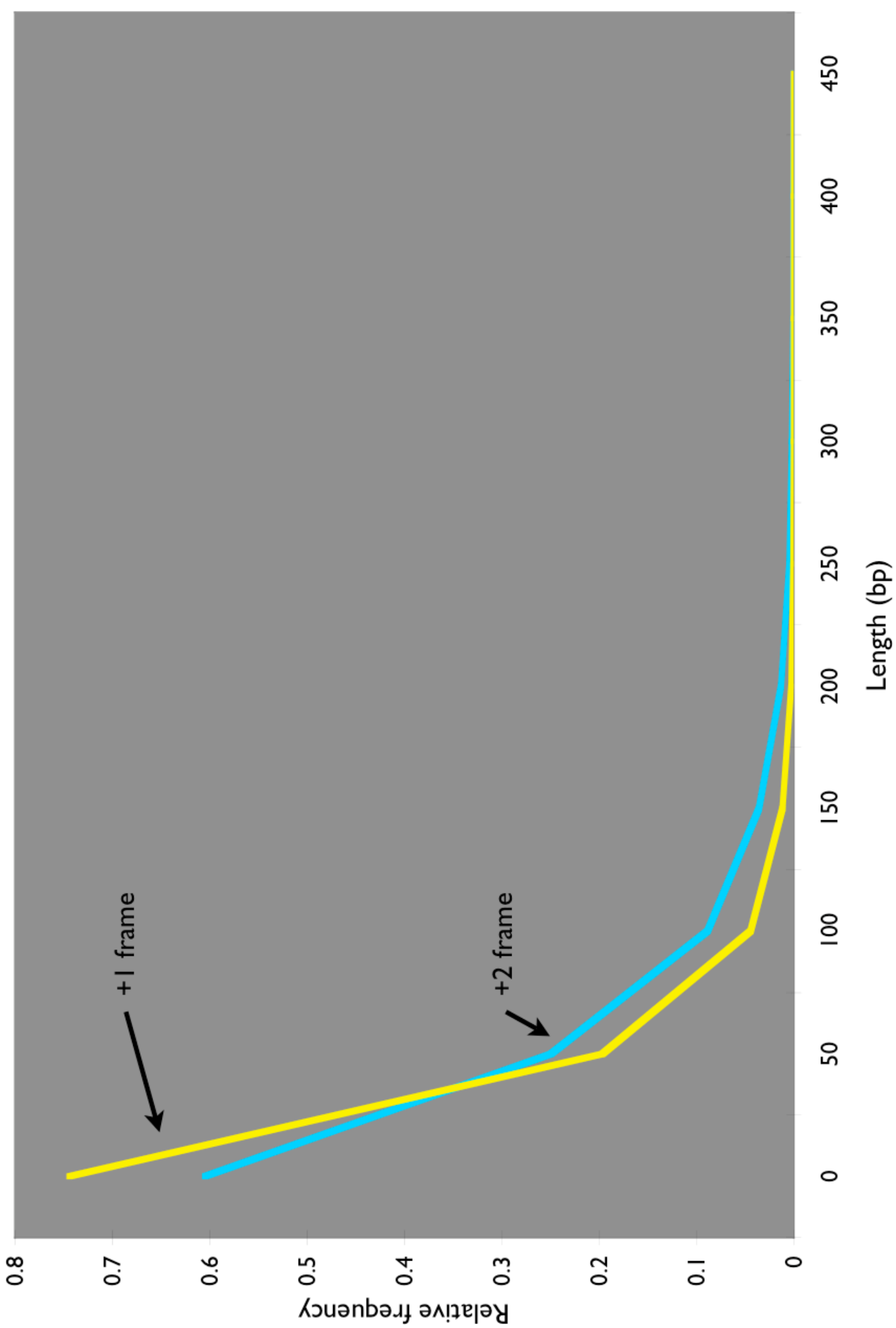

Figure S1. Distribution of lengths of maximal ARF detected in 10,000 simulated alignments.
