# Supplementary material for: A First Look at ARFome: Dual-Coding Genes in Mammalian Genomes
Source: PLoS Comput Biol. 2007 May 18;3(5):e91. doi: 10.1371/journal.pcbi.0030091 (PMC1868773; doi:10.1371/journal.pcbi.0030091)
Supplement: Supplementary file 2 [file pcbi.0030091.sg002.pdf]

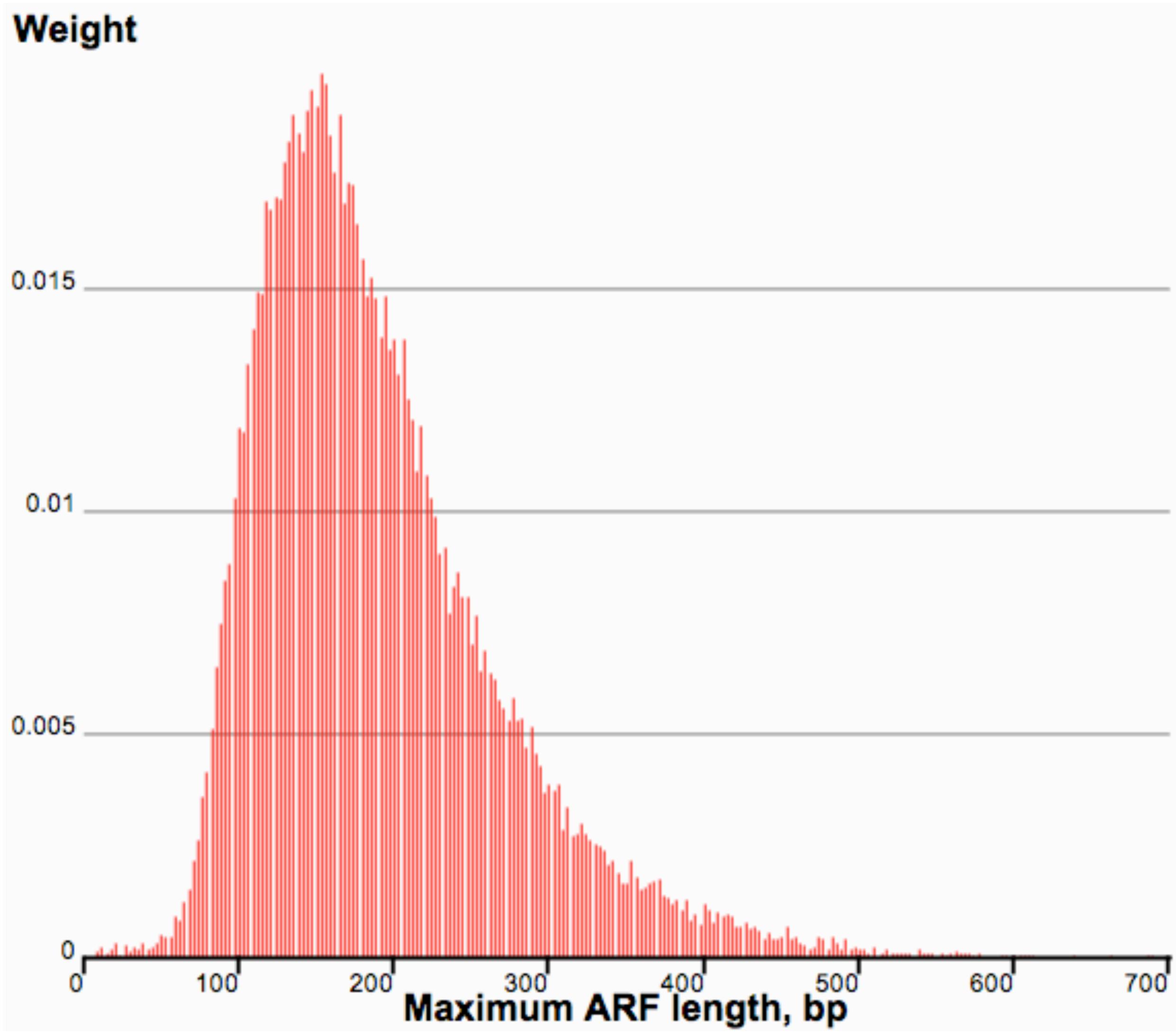

Figure S2. Distribution of lengths of maximal ARF, based on 35,000 parametric simulations based on codon model fits to orthologous gene alignments from 3 or 4 species (total of 39 gene fits, each with at least 500 bp sampled from equiprobably). Only 0.29% of simulated alignments had open ARF with 500 or more nucleotides.
