# Supplementary material for: A First Look at ARFome: Dual-Coding Genes in Mammalian Genomes
Source: PLoS Comput Biol. 2007 May 18;3(5):e91. doi: 10.1371/journal.pcbi.0030091 (PMC1868773; doi:10.1371/journal.pcbi.0030091)
Supplement: Supplementary file 3 [file pcbi.0030091.sg003.pdf]

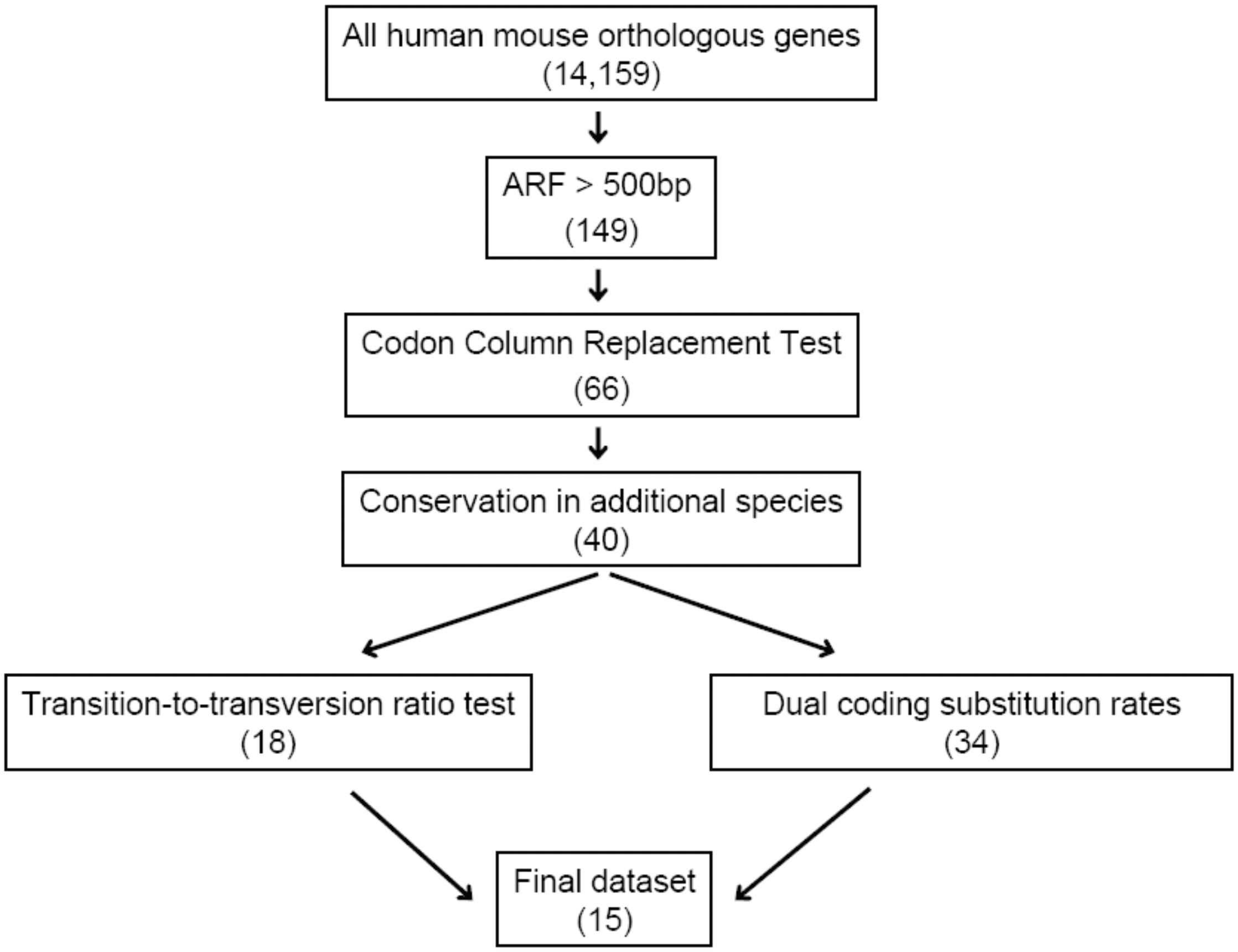

Figure S3. The number of possible dual coding genes (in parenthesis) and the corresponding criteria
