# Supplementary material for: A First Look at ARFome: Dual-Coding Genes in Mammalian Genomes
Source: PLoS Comput Biol. 2007 May 18;3(5):e91. doi: 10.1371/journal.pcbi.0030091 (PMC1868773; doi:10.1371/journal.pcbi.0030091)
Supplement: Supplementary file 4 [file pcbi.0030091.sg004.pdf]

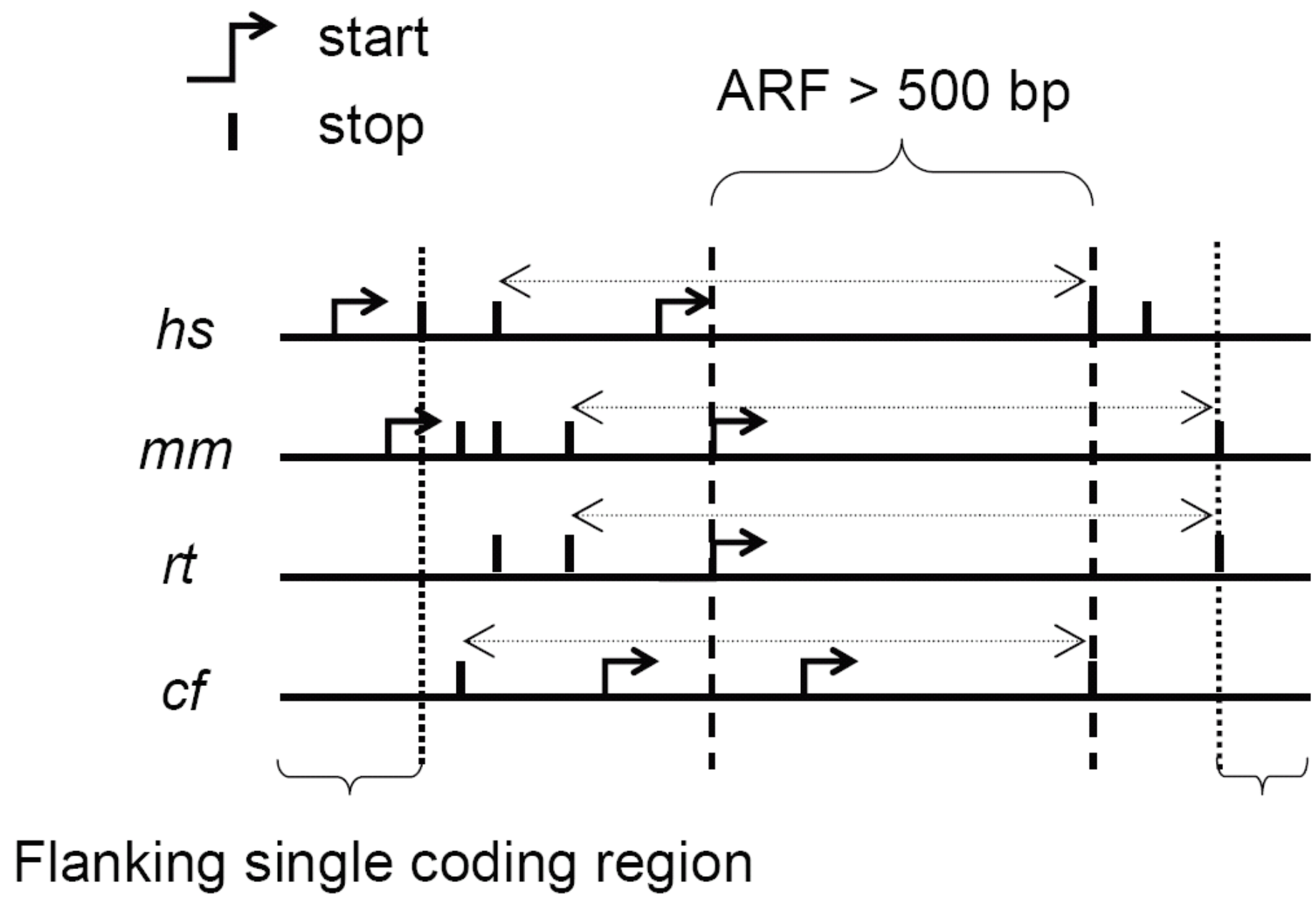

Figure S4. The finding and definition of conserved dual coding regions from multi species alignments. The orthologous transcripts from four species were first aligned and then translated using the second reading frame. Hence, additional start and stop codons appeared in the translation. For each of the species, an uninterrupted segment of peptides were identified (the dotted line with arrow ends at both direction) and the first start codon was marked. The region between the closest start-stop codons was defined as ARF region. From the same set of transcripts, regions from the beginning to first stop codon in any one of the species and the last stop codon to the end of the transcript were defined as flanking ORF region.
