# Supplementary material for: A First Look at ARFome: Dual-Coding Genes in Mammalian Genomes
Source: PLoS Comput Biol. 2007 May 18;3(5):e91. doi: 10.1371/journal.pcbi.0030091 (PMC1868773; doi:10.1371/journal.pcbi.0030091)
Supplement: Supplementary file 5 [file pcbi.0030091.st001.pdf]

Frame/Codon Position		SS	SN	NS	NN	Stop
F0	1	3.6	0.7	61.7	25.4	8.5
	2	0	0	4.0	87.2	8.8
	3	0	65.3	0	25.6	9.1
F1	1	0	0	4.0	88.2	7.8
	2	0	65.3	0	25.6	9.1
	3	3.6	0.7	61.0	25.1	9.6

Table S1. Proportion of substitution types (in %) in each codon position of F0 and F1 averaged over all possible nucleotide contexts.
