# Supplementary material for: A First Look at ARFome: Dual-Coding Genes in Mammalian Genomes
Source: PLoS Comput Biol. 2007 May 18;3(5):e91. doi: 10.1371/journal.pcbi.0030091 (PMC1868773; doi:10.1371/journal.pcbi.0030091)
Supplement: Supplementary file 6 [file pcbi.0030091.st002.pdf]

First/Second	A	C	G	T
A	AAA/86.89	ACA/86.89	AGA/86.89	ATA/41.31
	AAC/86.89	ACC/86.89	AGC/86.89	ATC/86.89
	AAG/86.89	ACG/86.89	AGG/86.89	ATG/64.10
	AAT/86.89	ACT/86.89	AGT/86.89	ATT/86.89
C	CAA/100.00	CCA/100.00	CGA/100.00	CTA/47.54
	CAC/100.00	CCC/100.00	CGC/100.00	CTC/100.00
	CAG/100.00	CCG/100.00	CGG/100.00	CTG/73.77
	CAT/100.00	CCT/100.00	CGT/100.00	CTT/100.00
G	GAA/93.44	GCA/93.44	GGA/93.44	GTA/44.42
	GAC/93.44	GCC/93.44	GGC/93.44	GTC/93.44
	GAG/93.44	GCG/93.44	GGG/93.44	GTG/68.93
	GAT/93.44	GCT/93.44	GGT/93.44	GTT/93.44
T	TAA/STOP	TCA/100.00	TGA/STOP	TTA/47.54
	TAC/100.00	TCC/100.00	TGC/100.00	TTC/100.00
	TAG/STOP	TCG/100.00	TGG/100.00	TTG/73.77
	TAT/100.00	TCT/100.00	TGT/100.00	TTT/100.00

Table S2. Proportion (%) of prefix and suffix codons (out of 3721 possibilities) which, for a given middle codon, do not induce a stop codon in the +1 reading frame. Brighter colors indicate less tolerated codons.
