# Supplementary material for: A First Look at ARFome: Dual-Coding Genes in Mammalian Genomes
Source: PLoS Comput Biol. 2007 May 18;3(5):e91. doi: 10.1371/journal.pcbi.0030091 (PMC1868773; doi:10.1371/journal.pcbi.0030091)
Supplement: Supplementary file 7 [file pcbi.0030091.st003.pdf]

#	gi #	Gene	RefSeq ID	GO categories
1	53831993	SF3A1	NM_005877	GO:0003723,GO:0000389,GO:0000398,GO:0006464,GO:0008380,GO:0005634, GO:0005681
2	4758467	GRP50	NM_004224	GO:0004872,GO:0008502,GO:0007165,GO:0007186,GO:0007267,GO:0005887, GO:0016020
3	4503680	FCGBP	NM_003890	
4	18201912	FOXN1	NM_003593	GO:0003700,GO:0003704,GO:0043565,GO:0006350,GO:0006357,GO:0006952, GO:0007275,GO:0008544,GO:0009887,GO:0005634,GO:0030216,GO:0050673,
5	27436942	RXRb	NM_021976	GO:0003700,GO:0003707,GO:0003713,GO:0004886,GO:0005496,GO:0005515, GO:0008270,GO:0016439,GO:0043565,GO:0046872,GO:0006350,GO:0006355, GO:0005634,GO:0005515
6	62954773	CSMD2	AB212622	GO:0016020,GO:0016021
7	31342353	ZNF598	NM_178167	GO:0003676,GO:0005515,GO:0008270,GO:0046872,GO:0005622
8	14165285	RHOBTB2	NM_015178	GO:0000166,GO:0005515,GO:0005525,GO:0007264,GO:0005622
9	24041034	NOTCH2	NM_024408	GO:0003706,GO:0004872,GO:0005509,GO:0005515,GO:0046982,GO:0001709, GO:0006350,GO:0006355,GO:0006916,GO:0006917,GO:0007050,GO:0007219, GO:0007399,GO:0008285,GO:0009887,GO:0016049,GO:0019827,GO:0030097, GO:0030154,GO:0046579,GO:0050793,GO:0005634,GO:0005887,GO:0009986, GO:0016020,GO:0005515,GO:0002011,GO:0030326,GO:0043065,GO:0007368, GO:0042060,
10	6513852	PCDH8	AF061573	GO:0005509,GO:0005515,GO:0007155,GO:0007156,GO:0007267,GO:0005886, GO:0005887,GO:0016331,GO:0001756
11	37655178	AP3B2	NM_004644	GO:0005215,GO:0005488,GO:0006892,GO:0006897,GO:0015031,GO:0005905, GO:0030137
12	109891936	DLGAP4	NM_014902	GO:0007267,GO:0016020
13	48762935	CSRP3	NM_003476	GO:0008270,GO:0046872,GO:0007519,GO:0030154,GO:0005634,GO:0005515, GO:0030018,GO:0002026,GO:0006874,GO:0048738
14	4758955	BZRAP1	NM_004758	GO:0030156,GO:0008150,GO:0005737,GO:0005739
15	48255896	SEMA6C	NM_030913	GO:0004872,GO:0007399,GO:0030154,GO:0016020,GO:0016021,GO:0007275
16	38348329	LANCL3	NM_198511	
17	52856410	CXXC1	NM_014593	GO:0003677,GO:0005515,GO:0008270,GO:0016563,GO:0045322,GO:0046872, GO:0006350,GO:0006355,GO:0005634,GO:0016607,GO:0016363
18	4557256	ADCY8	NM_001115	GO:0000287,GO:0008294,GO:0006171,GO:0007242,GO:0007611,GO:0005624, GO:0005886,GO:0016021
19	38176156	SPATA2	NM_006038	GO:0003674,GO:0007283,GO:0030154,GO:0005737
20	37537685	ZSCAN21	NM_145914	GO:0003700,GO:0008270,GO:0016563,GO:0046872,GO:0006350,GO:0006355, GO:0005622,GO:0005634,GO:0005634,GO:0003677
21	122114640	ZNF3	NM_032924	GO:0003676,GO:0008270,GO:0046872,GO:0006355,GO:0005622,GO:0005634, GO:0003700,GO:0006350,GO:0030154,GO:0045321
22	31317254	NLGN2	NM_020795	GO:0042043,GO:0007416,GO:0016337,GO:0045217,GO:0016021,GO:0045211, GO:0045202
23	58257667	KIAA0802	AB018345	
24	27436945	LMNA	NM_170707	GO:0005198,GO:0005515,GO:0005634,GO:0005882,GO:0005634,GO:0006998, GO:0005638
25	34147467	CCDC120	NM_033626	
26	28559070	DNMT3A	NM_175630	GO:0003677,GO:0003886,GO:0005515,GO:0008168,GO:0008270,GO:0016740, GO:0046872,GO:0006306,GO:0006349,GO:0000791,GO:0005634,GO:0005737, GO:0016363,GO:0003677,GO:0003886,GO:0008168,GO:0008270,GO:0016740, GO:0006306,GO:0006349,GO:0000791,GO:0005634,GO:0005737,GO:0016363, GO:0005515,GO:0000122,GO:0007283,GO:0003682,GO:0000792,GO:0006346, GO:0005720
27	13376631	ZC3H12A	NM_025079	
28	53832025	IQSEC2	NM_015075	GO:0005086,GO:0032012,GO:0005622NM_020235
29	18378730	BBX	NM_020235	

30	113423421	Predicted protein	XM_926122	
31	21071079	FBXL7	NM_012304	GO:0004842,GO:0005515,GO:0006511,GO:0000151
32	14017860	KIAA1822	AB058725	GO:0016021
33	6649056	TMEM2	AF137030	GO:0005044,GO:0001620
34	18379331	WAC	NM_100486	
35	113204605	RBAK	NM_021163	GO:0003676,GO:0008270,GO:0016564,GO:0046872,GO:0006355,GO:0005622, GO:0005634
36	117189905	MINK1	NM_153827	GO:0000166,GO:0004674,GO:0005083,GO:0005524,GO:0016740,GO:0006468, GO:0006950,GO:0007243,GO:0007254,GO:0007275,GO:0045060
37	52145308	LING01	NM_032808	GO:0016740
38	45433544	KIAA0460	NM_015203	
39	56790298	PSD	NM_002779	GO:0004871,GO:0005086,GO:0007165,GO:0032012,GO:0005575,GO:0005622
40	57165354	LPHN1	NM_001008701	GO:0004872,GO:0004930,GO:0005529,GO:0016524,GO:0007165,GO:0007218, GO:0016020,GO:0016021

Table S3. GO categories of the 40 candidate genes.
