# Supplementary material for: A First Look at ARFome: Dual-Coding Genes in Mammalian Genomes
Source: PLoS Comput Biol. 2007 May 18;3(5):e91. doi: 10.1371/journal.pcbi.0030091 (PMC1868773; doi:10.1371/journal.pcbi.0030091)
Supplement: Supplementary file 8 [file pcbi.0030091.st004.pdf]

#	gi #	Gene	Position
1	53831993	SF3A1	chr22:29066245-29068236
2	4758467	GRP50	chrX:150099804-150100352
3	4503680	FCGBP	chr19:45091552-45092458
4	18201912	FOXN1	chr17:23875883-23881964
5	27436942	RXRbeta	chr6:33274168-33276170
6	62954773	CSMD2	chr1:33862705-33874588
7	31342353	ZNF598	chr16:1991583-1993660
8	14165285	RHOBTB2	chr8:22918858-22920601
9	24041034	Notch2	chr1:120313786-120349540
10	6513852	PCDH8	chr13:52319345-52319863
11	37655178	AP3B2	chr15:81129664-81132688
12	109891936	DLGAP4	chr20:34493698-34494213
13	48762935	CSRP3	chr11:19160798-19170516
14	4758955	BZRAP1	chr17:53750639-53754726
15	48255896	SEMA6C	chr1:149372252-149373545
16	38348329	LANCL3	chrX:37316077-37316616
17	52856410	CXXC1	chr18:46063056-46064320
18	4557256	ADCY8	chr8:132121215-132121748
19	38176156	SPATA2	chr20:47955607-47956197
20	37537685	ZSCAN21	chr7:99499551-99500171
21	122114640	ZNF3	chr7:99506773-99507435
22	31317254	NLGN2	chr17:7259601-7260113
23	58257667	KIAA0802	chr18:8815386-8815898
24	27436945	LMNA	chr1:154371609-154372756
25	34147467	CCDC120	chrX:48811892-48812587
26	28559070	DNMT3A	chr2:25320271-25322437
27	13376631	ZC3H12A	chr1:37721192-37721704
28	53832025	IQSEC2	chrX:53280407-53282330
29	18378730	BBX	chr3:108992777-109006989
30	113423421		chr12:79179924-79185213
31	21071079	FBXL7	chr5:15981072-15981551
32	14017860	KIAA1822	chr14:99193155-99196459
33	6649056	TMEM2	chr9:73494904-73505558
34	18379331	WAC	chr10:28864495-28919728
35	113204605	RBAK	chr7:5071193-5071729
36	117189905	MINK1	chr17:4735561-4736830
37	52145308	LRRN6A	chr15:75693455-75693994

38	45433544	KIAA0460	chr1:148711467-148711997
39	56790298	PSD	chr10:104166187-104166726
40	57165354	LPHN1	chr19:14132416-14134627

Table S4. Genomic coordinates of the 40 candidate genes.
